# Psychometric validation of the Korean Hope-Action Inventory among university students

**DOI:** 10.3389/fpsyg.2026.1794159

**Published:** 2026-05-18

**Authors:** Sungsik Ahn, Hyung Joon Yoon

**Affiliations:** 1 Graduate School of Education, Keimyung University, Daegu, Republic of Korea; 2 Department of Learning and Performance Systems, The Pennsylvania State University, University Park, PA, United States

**Keywords:** confirmatory factor analysis, Hope-Action Inventory, Hope-Action Theory, human agency, psychometric validation, university students

## Abstract

The present study examined the psychometric properties of the Hope-Action Inventory (HAI) in Korean university students. Two independent samples (Sample 1: *N* = 2,096; Sample 2: *N* = 1,004) were used to compare competing models, including correlated seven-factor, hierarchical, and bifactor structures, and to evaluate reliability and convergent validity. Across both samples, a modified seven-factor correlated model demonstrated the best overall fit among the tested models. Internal consistency was examined using Cronbach’s α and McDonald’s ω, indicating excellent reliability for the total score and acceptable to good reliability for all subscales. Convergent validity was supported by expected associations between HAI scores and theoretically related constructs, including vocational identity, components of human agency, career goal and preparation competencies, dispositional hope, optimism, flexibility-related measures, and career decision-making difficulties. At the total-score level, the HAI showed strong positive correlations with adaptive career-related constructs and a moderate negative correlation with career decision-making difficulties. Overall, the findings provide evidence supporting the reliability and validity of the Korean HAI for use in career research and practice.

## 1 Introduction

The career environment in the 21st century is characterized by a VUCA (Volatility, Uncertainty, Complexity, Ambiguity) context (Bennett and Lemoine, 2014), shaped by rapid digitalization and structural transformations in the labor market. In this environment, individuals are increasingly required to continuously adapt to change across the life span, rather than make a single career choice at a fixed point in time (Savickas et al., 2009; Niles et al., 2014). For young people in particular, competencies such as career adaptability, self-directed career management, and effective coping with transitions have become essential (OECD, 2019, 2023). Central to these competencies is human agency—the ability to intentionally influence one’s functioning and life circumstances (Bandura, 2001).

Hope-Action Theory (HAT; Niles et al., 2019) provides an agentic framework for understanding career development in contexts of uncertainty and change. Grounded in Bandura’s (2001) concept of human agency, HAT conceptualizes hope as an action-oriented process that enables individuals to set meaningful career goals, implement plans, and adapt to evolving circumstances (Niles et al., 2014). The theory integrates three perspectives: Bandura’s (2001) social cognitive theory of agency, Snyder’s (2002) hope theory emphasizing agency and pathways thinking, and Hall’s (1996) protean career perspective, specifically the career metacompetencies of self-identity and adaptability. Building on these foundations, HAT aims to support individuals in actively constructing their careers and responding adaptively to the volatility, uncertainty, complexity, and ambiguity of the 21st-century labor market. More specifically, HAT aims to explain the cognitive and behavioral processes by which individuals, grounded in human agency, cultivate action-oriented hope to set meaningful career goals, develop pathways, and adapt to uncertainties in volatile labor markets (Niles et al., 2014). The key processes represented in HAT are reflected in the seven dimensions assessed by the Hope-Action Inventory (HAI), as summarized in Table 1.

**TABLE 1:** Conceptual mapping of Hope-Action Inventory dimensions to components of Hope-Action Theory.

Dimension	Theoretical component	Conceptual description
Hopefulness	The central hope component in HAT; Different from Snyder’s hope constructs. Close to the general public’s understanding of hope	Positive expectancy about one’s future
Self-reflection	Self-reflectiveness (Bandura, 2001)	Introspective behaviors about the self and the past
Self-clarity	Identity metacompetency (Hall, 1996)	Being clear about one’s identity and strengths
Visioning	Pathways thinking (Snyder, 2002) and forethought (Bandura, 2001)	Envisioning future possibilities
Goal setting and planning	Goals and intentionality	Setting goals and planning a course of action
Implementing	Agency thinking and self-reactiveness	Executing plans
Adapting	Adaptability metacompetency	Adjusting plans and actions when faced with changes

The mapping reflects the theoretical integration underlying Hope-Action Theory (HAT), which draws on Bandura’s (2001) Human Agency Theory, Snyder’s (2002) hope theory, and Hall’s (1996) career metacompetencies in his Protean Career Theory.

Hope-Action Theory proposes a multidimensional structure in which multiple action competencies interact dynamically around hope (Niles et al., 2010, 2014). This structure has been represented through a pinwheel model (Niles et al., 2019; Yoon, 2020), in which hopefulness occupies the central position and six core hope-action career competencies: self-reflection, self-clarity, visioning, goal setting and planning, implementing, and adapting. The HAI was developed to operationalize this model and to assess its dimensions in research and applied settings (Niles et al., 2010; Yoon et al., 2015, 2019).

Empirical studies have suggested that the HAI functions effectively in diverse applied settings. Higher HAI scores have been consistently associated with more proactive career-development engagement, including the adoption of new job-search perspectives, strengthened career planning, and increased confidence in career decision-making (Amundson et al., 2018; Clarke et al., 2018; Niles et al., 2014; Yoon et al., 2015, 2019). These findings suggest that the HAI captures meaningful competencies relevant to adaptive career behavior.

### 1.1 Evolution of the instrument

The HAI evolved from earlier work on the Hope-Centered Career Inventory (HCCI), which was originally developed to operationalize the core competencies proposed in the Hope-Centered Model of Career Development (HCMCD; Niles et al., 2010). The HCCI assessed seven competencies supporting effective career self-management: hope, self-reflection, self-clarity, visioning, goal setting and planning, implementing, and adapting.

Subsequent work refined the measurement of these competencies to improve psychometric performance. In particular, Yoon (2017) expanded the item pool and conducted psychometric analyses that resulted in the refinement of the item pool, resulting in the reduction of the instrument from 42 items to 28 items to improve conceptual clarity and reliability, particularly in dimensions that had demonstrated weaker internal consistency in earlier studies. Although the revised item set was developed at this stage, the instrument continued to be referred to as the HCCI in the literature, which is labeled “Revised HCCI” in Table 2.

**TABLE 2:** Evolution of the Hope-Action Inventory (HAI).

Instrument name	Corresponding theory name	Key characteristics	Representative studies
Career flow index	Hope-Centered Model of Career Development	Original instrument developed to operationalize hope-centered career competencies related to career development and transitions. The initial instrument contained a larger pool of items representing the competency domains.	Niles et al. (2010)
Hope-Centered Career Inventory (HCCI)	Hope-Centered Model of Career Development	Early validation studies conducted across different cultural contexts using the original HCCI item set. Results generally supported a multidimensional structure while indicating variability in some subscales across samples.	Amundson et al. (2018); Clarke et al. (2018); Schreiber et al. (2013); Santilli et al. (2021); Yoon et al. (2015)
Revised HCCI	Hope-Centered Model of Career Development	Item pool expanded and refined, followed by item reduction from 42 items to 28 items to improve psychometric performance and conceptual clarity. The revision particularly improved reliability in dimensions that had shown weaker performance, such as Self-reflection. Although the item set was revised, the instrument was still referred to as HCCI at this stage.	Yoon (2017)
Hope-Action Inventory (HAI)	Hope-Action Theory (Theory renamed)	The revised 28-item instrument (Revised HCCI) was formally presented under the name Hope-Action Inventory, aligning the instrument with the articulation of Hope-Action Theory and its emphasis on action-oriented career hope competencies.	Yoon et al. (2019)
HAI	Hope-Action Theory	Subsequent psychometric studies examined the factor structure and reliability of the HAI across diverse populations and contexts.	Currie et al. (2024); Yoon et al. (2026); current Korean validation study

As the theoretical framework underlying the instrument further evolved into Hope-Action Theory, the revised 28-item instrument was subsequently introduced under the name Hope-Action Inventory (HAI) while retaining the same seven conceptual competency domains (Yoon et al., 2019; Niles et al., 2019). More recently, the HAI has been examined in additional validation studies conducted in Canada and other contexts (Currie et al., 2024; Yoon et al., 2026). The present study also employs the 28-item version of the HAI. The developmental relationship among these versions is summarized in Table 2.

### 1.2 Factor structure of the HAI

Previous validation studies have examined different versions of the instrument across diverse cultural contexts. In a study with German adult samples using HCCI, the hierarchical factor structure was generally supported; however, the second-order loading from the higher-order hope-action competency factor to the Self-reflection factor was relatively low (0.37), indicating that Self-reflection contributed less to the overarching construct compared with other dimensions (Schreiber et al., 2013). This pattern may reflect potential cultural and linguistic differences in the understanding of self-reflection items rather than a fundamental flaw in the hierarchical model. In a study with Italian healthcare workers, also based on the HCCI, the seven-factor correlated model demonstrated better fit than the hierarchical model (Santilli et al., 2021), suggesting that the action competencies may function as relatively independent but related dimensions rather than as clear indicators of a single overarching hope-action construct in certain cultural contexts. In contrast, a recent Canadian study using the HAI item set, which examined only the hierarchical structure, supported a hierarchical factor model in a clinical sample (Currie et al., 2024).

Taken together, previous studies have supported different structural representations of the same underlying theoretical model rather than converging on a single psychometric representation. This suggests that the key issue may not be whether the HAI is multidimensional, but how the relationships among the hope-action competencies should be represented structurally. Accordingly, it is important to compare alternative structural models—including correlated, hierarchical, and bifactor approaches—to determine which representation best reflects the theoretical organization of the hope-action competencies. From a measurement perspective, these models represent different ways of conceptualizing multidimensional constructs (Brown, 2015). A correlated factor model treats the competencies as related but distinct dimensions. In contrast, a hierarchical (second-order) model assumes that the correlations among competencies are explained by a higher-order construct (Chen et al., 2005). A bifactor model, however, specifies a general factor that accounts for common variance across all items while specific factors account for additional domain-specific variance (Reise, 2012; Rodriguez et al., 2016). Therefore, comparing these models allows researchers to examine how the theoretically defined competencies are organized psychometrically.

Notably, the German and Italian studies were based on the HCCI, the earlier version of HAI, whereas the Canadian study (Currie et al., 2024) and the present research employ the HAI, in which seven items were refined to enhance conceptual clarity. Given these version differences and the differing structural representations reported in previous studies, it is critical to examine how the theoretically specified structure is represented psychometrically across cultural contexts. In particular, empirical evidence is needed to determine whether the factor structure supported in the Canadian clinical sample can be replicated among Korean university students.

Although previous studies have reported different psychometric representations of the HAI, these variations do not indicate inconsistency in the underlying theoretical structure. The HAI was developed as a theory-driven instrument based on Hope-Action Theory, which specifies seven core competencies of intentional career development. Therefore, the purpose of the present study was not to explore a new factor structure, but to examine how the theoretically specified competencies are best represented structurally—whether as correlated factors, a hierarchical structure, or a bifactor structure—in a Korean context. Because the factor structure was specified a priori, confirmatory factor analysis was considered more appropriate than an exploratory approach, as CFA is designed to evaluate hypothesized factor structures, whereas EFA is typically used when the underlying structure is unknown (Brown, 2015; Kline, 2016). Accordingly, the present study compared multiple theoretically grounded CFA models to examine the structural representation of the HAI rather than conducting an exploratory factor analysis.

### 1.3 Internal consistency of HAI

Across previous studies, the HAI has consistently demonstrated high internal consistency at the total-score level, with Cronbach’s α ranging from 0.91 to 0.94 in North American samples (Clarke et al., 2018; Amundson et al., 2018; Yoon et al., 2015, 2026). Subscale reliabilities have generally been acceptable to good, particularly for Self-clarity (α = 0.69–0.82), Visioning (α = 0.75–0.86), Goal Setting and Planning (α = 0.74–0.87), Implementing (α = 0.77–0.85), and Adapting (α = 0.72–0.81). However, Self-reflection has shown relatively lower and more variable reliability across studies (α = 0.54–0.78), suggesting potential sensitivity of this dimension to contextual or linguistic interpretation. Similar variability has been observed for the Hopefulness subscale (α = 0.63–0.87), depending on sample characteristics and intervention context. In applied intervention studies such as the Hope to Work program with refugees, overall reliability remained strong (α = 0.84–0.92; Yoon et al., 2019), indicating that the instrument performs robustly in practice despite subscale-level fluctuations.

Cross-cultural validation studies have reported generally adequate reliability of the HAI total score in diverse contexts, with α = 0.88 in Germany (Schreiber et al., 2013), α = 0.87 in Italy (Santilli et al., 2021), and α = 0.88 in Canada using the HAI (Currie et al., 2024). Subscale reliabilities, however, showed greater variability across countries and item versions. In the German and Italian studies, most subscales demonstrated acceptable reliability—particularly Self-clarity (α = 0.65–0.77), Visioning (α = 0.75–0.82), and Goal Setting and Planning (α = 0.71–0.75)—whereas Self-reflection consistently yielded lower coefficients (α = 0.59 in Germany; α = 0.60 in Italy), again pointing to potential cultural or linguistic sensitivity of this factor.

In contrast, the Canadian study employing the HAI items reported uniformly high reliabilities across all subscales (α = 0.90–0.92), including Self-reflection (α = 0.92), suggesting that the item revisions may have enhanced the clarity and coherence of this dimension (Currie et al., 2024). These findings imply that earlier cross-cultural variability in Self-reflection may have reflected item-level limitations rather than conceptual weaknesses, and underscore the need to re-examine the psychometric properties of the HAI. Therefore, empirical validation of the HAI in Korean samples is required to determine whether the improved reliability observed in Canada can be replicated and to clarify how the factor structure operates in a non–North American context.

### 1.4 Convergent validity of HAI

Previous studies have supported the convergent validity of the HAI through associations with theoretically related constructs. In U.S. college samples, the HAI total score correlated strongly with the Assessment of Human Agency (Yoon, 2011, *r* = 0.82) and the Adult Hope Scale (Snyder et al., 1991, *r* = 0.74), and moderately with vocational identity (Holland et al., 1980, *r* = 0.45) (Niles et al., 2010). The Hopefulness subscale showed conceptually consistent relations with the Adult Hope Scale (Snyder et al., 1991, *r* = 0.65), the Assessment of Human Agency (Yoon, 2011, *r* = 0.55), and Vocational Identity scale (Holland et al., 1980, *r* = 0.41) (Yoon, 2011). Italian data likewise demonstrated moderate convergence with the Adult Hope Scale (Snyder et al., 1991; *r* = 0.20–0.54), particularly for the HAI total score (*r* = 0.54) and the Hopefulness (*r* = 0.50) and Implementing (*r* = 0.47) subscales. Associations with the Satisfaction with Life Scale (Diener et al., 1985) were more modest (*r* = 0.15–0.40), with the strongest relation observed for the Hopefulness subscale (*r* = 0.40) (Santilli et al., 2021). A recent Canadian study further demonstrated robust validity of the HAI (Currie et al., 2024), revealing a strong positive correlation with the Adult Hope Scale (Snyder et al., 1991; *r* = 0.76) and moderate negative associations with measures of hopelessness and pessimism, ranging from *r* = −0.44 to −0.64. Specifically, correlations were strongest with the State Hopelessness Scale (Dunn et al., 2014; *r* = −0.64) and Trait Hopelessness Scale (Dunn et al., 2014; *r* = −0.60), followed by the Brief-H-Neg scale (Fraser et al., 2014; *r* = −0.51) and the Life Orientation Test-Revised (LOT-R; Scheier et al., 1994) Pessimism subscale (*r* = −0.44), suggesting that the HAI reflects an action-oriented form of hope distinct from general negative expectancy.

Nevertheless, these studies have primarily examined convergent validity at the level of the HAI total score, with relatively limited attention to the differential validity of individual subscales. Given that HAT conceptualizes hope-action competencies as a multidimensional construct comprising distinct yet interrelated components, evidence based solely on total scores may obscure meaningful patterns at the subscale level. The present study therefore aims to extend previous research by evaluating convergent validity for both the total score and specific HAI dimensions. It is hypothesized that theoretically matched measures will show stronger associations with corresponding subscales, thereby providing more fine-grained evidence for the construct validity of the HAI.

Specifically, convergent validity hypotheses were derived from the conceptual alignment between each external measure and specific components of the HAI. The Vocational Identity scale (VI; Holland et al., 1980) was expected to correlate most strongly with the HAI Self-Clarity subscale, because the VI captures the clarity and stability of the career self-concept, which conceptually parallels the self-understanding represented in Self-Clarity. The Assessment of Human Agency (AHA; Yoon, 2011) was expected to show differentiated associations with the HAI subscales—intentionality with Goal Setting and Planning, forethought with Visioning, self-reactiveness with Implementing, and self-reflectiveness with Self-reflection—because both instruments assess parallel agentic processes grounded in Bandura’s (2001) Human Agency Theory. The Adult Hope Scale (Snyder et al., 1991) and the Life Orientation Test-Revised (Scheier et al., 1994) were expected to correlate most strongly with the HAI Hopefulness subscale, as these measures capture dispositional hope and generalized positive expectancy. The career goal setting and preparation subscale of the College Students’ Career Competencies Scale (Jeong, 2015) was expected to relate primarily to the HAI Goal Setting and Planning and Implementing dimensions, reflecting their shared focus on concrete planning and enactment. Flexibility subscales from the Vocational Identity Status Assessment (Porfeli et al., 2011) and the Planned Happenstance Career Inventory (Kim et al., 2014) were expected to converge with the HAI Adapting dimension, given their common emphasis on openness to change. Finally, the Emotional and Personality-Related Career Decision-Making Difficulties–Short Form (Gati et al., 2011) was expected to show negative associations with the HAI total score, as it assesses barriers theoretically opposite to hope-action competencies.

### 1.5 The Korean context and rationale for validation

In addition to these theoretical considerations, the Korean context provides a meaningful setting for examining hope-action competencies. Although South Korea maintains one of the highest rates of tertiary education attainment among OECD countries, the transition from university to stable employment has become increasingly prolonged and competitive for young adults (OECD, 2019, 2023). Recent cohort analyses indicate that school-to-work transitions among Korean young adults have become more delayed and unstable compared with earlier generations, reflecting structural challenges in the youth labor market (Byun, 2025). Within this environment, empirical research has consistently emphasized the importance of proactive career behaviors and psychological career resources among Korean university students. For example, career preparation behavior has been shown to be positively associated with academic self-efficacy and career decision level (Kim and Ra, 2022), and it is positively related to career satisfaction during the university-to-work transition (Ra and Kim, 2023).

Furthermore, career adaptability has been identified as a psychosocial resource that supports students’ capacity to cope with career-related challenges and facilitates career decision-making self-efficacy (Lee and Jung, 2022). Recent research on Korean university students has also suggested the potential relevance of examining vocational identity status together with hope-related action resources when designing differentiated career interventions (Ahn, 2025). Taken together, these findings highlight the importance of action-oriented career competencies—such as proactive career preparation, career decision-related self-efficacy, and adaptability—in the Korean context. Examining whether the Hope-Action Inventory (HAI) validly captures these competencies among Korean university students is therefore an important step in evaluating the applicability of Hope-Action Theory in this context.

The Korean version of the HAI is intended to be used in both counseling practice and broader applied contexts. In university career centers and counseling services, the HAI may support the assessment of hope-action competencies by identifying students’ strengths and developmental needs in areas such as self-reflection, goal setting, and adaptability. Such information may assist practitioners in providing more targeted career guidance and in addressing students’ career decision-making difficulties. The Korean HAI may serve as a useful tool for research and program evaluation in career development initiatives designed to support young adults’ transition from education to work. In this way, the instrument may contribute to a better understanding of hope-action competencies among Korean university students and provide a foundation for developing evidence-informed career interventions and programs in the Korean context.

### 1.6 The present study

The primary aim of this study is to evaluate the psychometric properties of the Korean version of the HAI in university students, with particular attention to its factorial structure, internal consistency, test-retest reliability, and convergent validity. Three specific hypotheses guide the structural investigation:

H1: The seven-factor correlated model is expected to provide the best overall fit among the competing structural representations, consistent with the theoretically multidimensional nature of hope-action competencies and the previous findings (Santilli et al., 2021).

H2: The hierarchical model is expected to demonstrate acceptable overall fit but comparatively weaker fit than the correlated model, reflecting the mixed findings in previous studies (Currie et al., 2024; Schreiber et al., 2013).

H3: The bifactor model is expected to provide evidence of a general hope-action factor coexisting with distinct specific competencies, although it is not expected to outperform the correlated model in overall model fit.

Convergent validity was examined using measures of constructs theoretically related to hope-action competencies. These measures were selected based on their relevance to Hope-Aaction Theory and their use in prior validation studies of the HAI (Currie et al., 2024; Niles et al., 2010; Santilli et al., 2021), including vocational identity, human agency, dispositional hope, optimism, career goal and preparation competency, flexibility, and career decision-making difficulties.

## 2 Materials and methods

### 2.1 Participants

The data for this study were obtained from two independent student needs surveys conducted by the career centers of two 4-year private universities in Seoul. Both surveys were administered by the respective institutions as part of routine administrative efforts to assess students’ career-related needs, and the research team was provided with the resulting datasets for secondary analysis.

The two datasets served distinct analytic purposes within the study design. For Sample 1, the research team was initially provided with a dataset consisting of 2,220 responses. To ensure data quality, 124 cases were excluded due to incomplete data or evidence of careless responding (e.g., patterned responses or lack of response variability). This resulted in a final analytic sample of 2,096 participants for the structural validation of the scale. Sample 1 (*N* = 2,096) was used to examine the factorial structure and internal consistency of the Korean version of the HAI. Sample 1 included only HAI items and was therefore suitable for confirmatory factor analysis and reliability estimation.

For Sample 2, 1,129 students successfully accessed and initiated the survey. Following the exclusion of 125 cases identified as incomplete or showcasing careless responding patterns (e.g., straight-lining), the final sample size was 1,004. Sample 2 (*N* = 1,004) was used as an independent validation sample to cross-validate the modified factor structure derived from Sample 1 and to examine external validity. In addition to the HAI, Sample 2 included multiple theoretically related career and psychological measures, allowing for correlational analyses.

Participants in Sample 1 consisted of 1,291 male students (61.6%) and 805 female students (38.4%), representing all undergraduate grade levels and a wide range of academic fields, including natural sciences and engineering (55.7%), humanities and social sciences (37.0%), arts and physical education (4.0%), and medicine and nursing (3.3%). Participants in Sample 2 included 569 male students (56.7%) and 435 female students (43.3%), with a distribution across grade levels and academic fields comparable to that of Sample 1. Across both datasets, the majority of participants were currently enrolled students, reflecting typical undergraduate populations at 4-year universities in Korea.

To examine the test-retest reliability of the HAI, Sample 3 (*N* = 50) was recruited using convenience sampling. Participants were undergraduate students enrolled in a teacher education course taught by one of the researchers. To minimize potential confounding effects, the selected course did not include any career intervention components. Participation was voluntary and had no impact on course standing or grades. As an incentive, a small amount of extra course credit was provided upon completion of both the initial and follow-up assessments conducted over a 4-week interval. Of the 73 students who initially participated, 50 students (68.5%) completed both assessments and were included in the final analysis. The sample was predominantly female (94.0%) and primarily composed of students affiliated with colleges of education (58.0%).

### 2.2 Procedure and data acquisition

To support the practical utility and psychometric rigor of the institutional surveys, the research team provided technical consultation to the career centers during the instrument development phase. This collaborative effort ensured that the survey items, including the HAI, were aligned with established career development theories to accurately assess student needs. While the career centers maintained full ownership of the data collection process for administrative purposes, the research team assisted in establishing the scoring protocols and calculation methods for the institutional reports. Following the completion of the internal assessments, de-identified datasets were shared with the research team for secondary scholarly analysis, as pre-planned to explore the structural integrity of measures in the Korean context.

### 2.3 Instruments

#### 2.3.1 Hope-Action Inventory (HAI)

The present study employed the Hope-Action Inventory (HAI; Niles et al., 2010, 2014; Yoon, 2017, Yoon et al., 2019) to evaluate its psychometric properties with Korean university students. The original English version was translated into Korean following established guidelines for the cross-cultural adaptation of self-report measures (Beaton et al., 2000). Forward translation was conducted by a bilingual doctoral student in counseling, followed by an independent back-translation by another bilingual doctoral student. Discrepancies between versions were resolved through iterative cycles of translation and back-translation and researcher discussion until full semantic equivalence was achieved. The scale consists of 28 items assessing core dimensions of hope-action competency, including Hopefulness, Self-Reflection, Self-Clarity, Visioning, Goal Setting and Planning, Implementing, and Adapting. Items are rated on a 4-point Likert scale, with higher scores indicating stronger levels of hope-action competency. Previous studies have reported acceptable internal consistency for the total scale and subscales (α = 0.71–0.92; Niles et al., 2014; Santilli et al., 2021; Currie et al., 2024; Yoon et al., 2026). The HAI is a proprietary instrument, and the full item set cannot be reproduced here. However, Supplementary Appendix A provides representative English and Korean item examples presented side by side (three items per subscale) to illustrate the translation fidelity and face validity of the Korean adaptation.

#### 2.3.2 Vocational Identity scale (VI)

Vocational identity was assessed using the Vocational Identity (VI) scale from *My Vocational Situation* (Holland et al., 1980), which measures the clarity and stability of the career self-concept. The scale comprises 18 dichotomous items (*true/false*), with vocational-confusion items reverse-coded so that higher scores indicate stronger vocational identity. The original instrument demonstrated strong internal consistency (0.86–0.89), and the Korean version validated by Kim T.-S. (2003) also showed acceptable reliability (Cronbach’s α = 0.77). In the present study, the VI was expected to correlate most strongly with the HAI Self-Clarity subscale, given their shared focus on coherence and confidence in career self-understanding. An example item is: “I need reassurance that I have made the right choice of occupation” (reverse-coded; true/false).

#### 2.3.3 Assessment of Human Agency (AHA)

The Assessment of Human Agency (AHA) was developed by Yoon (2011) to operationalize Bandura’s concept of human agency, which constitutes one of the core theoretical foundations of Hope-Action Theory (HAT) alongside Snyder’s hope theory and the protean career perspective. The AHA measures human agency as defined by Bandura (2001) as the capacity to exercise control over one’s life. The scale comprises 12 items assessing four components—intentionality, forethought, self-reactiveness, and self-reflectiveness—rated on a four-point Likert scale. Yoon (2011) reported high internal consistency (Cronbach’s α = 0.88–0.90). The Korean validation by Ahn (2022) also showed good reliability (α = 0.88; subscales α = 0.63–0.77). The AHA was used to examine convergent validity of the HAI, with expected correspondence between intentionality–Goal Setting and Planning, forethought–Visioning, self-reactiveness–Implementing, and self-reflectiveness–Self-reflection. An example item is: “I have end results in mind before I begin something” (four-point Likert).

#### 2.3.4 Dispositional Hope Scale (K-DHS)

Trait hope was assessed using the Korean Dispositional Hope Scale (K-DHS; Choi et al., 2008), based on the original instrument by Snyder et al. (1991). The scale consists of 12 items, including four filler items, rated on a five-point Likert scale, and measures two components of hope—agency and pathways thinking. Previous research reported acceptable internal consistency (Cronbach’s α = 0.74–0.84; Snyder et al., 1991), and the Korean version also demonstrated good reliability (α = 0.82; Choi et al., 2008). In the present study, the K-DHS total score was used to examine convergent validity, and it was expected to correlate most strongly with the Hopefulness subscale of the HAI. An example item is: “I energetically pursue my goals” (five-point Likert).

#### 2.3.5 Life orientation Test-Revised (LOT-R)

Dispositional optimism was measured using the Life Orientation Test-Revised (LOT-R; Scheier et al., 1994; Korean version: Kim S. H., 2003). The scale consists of six scored items and four filler items rated on a five-point Likert scale, with higher scores indicating greater optimism. Previous research reported acceptable internal consistency (Cronbach’s α = 0.78; Scheier et al., 1994), and the Korean version also demonstrated comparable reliability (α = 0.71; Choi, 2008). Prior studies have shown significant positive correlations between optimism and hope (*r* = 0.50–0.60; Gibb, 1990; Holleran and Snyder, 1990). In the present study, LOT-R scores were expected to converge most strongly with the Hopefulness subscale of the HAI. An example item is: “In uncertain times, I usually expect the best” (five-point Likert).

#### 2.3.6 College Students’ Career Competencies Scale-Goal (CCS-Goal)

From the College Students’ Career Competencies Scale (CCS; Jeong, 2015), the career goal setting and preparation subscale (CCS-Goal) was used. The CCS-Goal has four items rated on a 5-point Likert scale, with higher scores indicating stronger competency in career goal setting and preparation. The original validation study reported good internal consistency (Cronbach’s α = 0.82; Jeong, 2015). This subscale was used to examine convergent validity, and the strongest associations were expected with the Goal Setting and Planning and Implementing dimensions of the HAI. An example item is: “I manage my time carefully to achieve my career goals” (five-point Likert).

#### 2.3.7 Vocational Identity Status Assessment-flexibility (VISA-Flex)

From the Vocational Identity Status Assessment–Korean version [VISA-K; Lee et al., 2014; based on Porfeli et al. (2011)], the career flexibility subscale (VISA-Flex) was used. This subscale consists of four items rated on a five-point Likert scale, with higher scores indicating greater openness to revising career commitments and adapting to changing circumstances. Previous research demonstrated adequate internal consistency [α = 0.81–0.83 in Porfeli et al. (2011); α = 0.86 in Lee et al. (2014)]. The VISA-Flex was included to examine convergent validity, and the strongest associations were expected with the Adapting dimension of the HAI. An example item is: “I will probably change my career goals” (five-point Likert).

#### 2.3.8 Planned Happenstance Career Inventory-flexibility (PHCI-Flex)

From the Planned Happenstance Career Inventory (PHCI; Kim et al., 2014), the flexibility subscale (PHCI-Flex) was used. This subscale consists of five items rated on a five-point Likert scale, with higher scores indicating greater willingness to revise attitudes and adapt to unplanned career opportunities. The original development study reported adequate internal consistency (α = 0.82–0.84; Kim et al., 2014). The PHCI-Flex was included to examine convergent validity, and the strongest associations were expected with the Adapting dimension of the HAI. An example item is: “I am flexible about considering multiple options rather than pursuing only one career path” (five-point Likert).

#### 2.3.9 Emotional and Personality-Related Career Decision-Making Difficulties-Short Form (EPCD-SF)

Career decision-making difficulties were measured using the Emotional and Personality-Related Career Decision-Making Difficulties-Short Form (EPCD-SF; Gati et al., 2011; Korean version: Jin et al., 2015). The original scale has 25 items which are rated on a nine-point Likert scale, with higher scores indicating greater emotional and personality-related barriers to career decision making. In the Korean validation study, two items were removed, resulting in 23 items, and the adapted version demonstrated excellent internal consistency (α = 0.92; Jin et al., 2015). The EPCD-SF was included to examine convergent validity, and negative associations were expected with the HAI total score. An example item is: “I can’t find out enough about all the occupations to make the right choice” (nine-point Likert).

### 2.4 Data analysis

Analyses were conducted in a staged manner to evaluate the factorial structure and psychometric properties of the Korean HAI. Step 1 used Sample 1 to test the original measurement structure through confirmatory factor analysis (CFA). A CFA-first approach was adopted rather than an exploratory one, as the HAI is a theory-driven instrument whose seven-factor structure is theoretically specified within Hope-Action Theory and has been examined in prior validation studies (Currie et al., 2024; Niles et al., 2010). Because the Korean adaptation retained the original item content and conceptual structure, the purpose of the analysis was to examine whether the theoretically specified structure could be replicated in a new cultural context rather than to derive a new factor structure In cross-cultural validation research, confirmatory approaches are typically preferred when the measurement model is theoretically defined in advance, as they allow for direct evaluation of model generalizability while preserving conceptual consistency across contexts (Brown, 2015; Kline, 2016).

An exploratory factor analytic (EFA) approach was therefore not adopted because modifying item composition or factor membership may compromise cross-cultural comparability and limit the interpretability of cumulative findings across studies. The HAI was developed as a theoretically specified instrument with fixed item–factor relationships grounded in Hope-Action Theory; thus, preserving the original item structure is essential for maintaining conceptual and measurement equivalence. Allowing item reconfiguration through EFA could alter the meaning of the underlying competencies and shift the focus from theory testing to scale redevelopment.

To reduce the possibility of sample-specific overfitting, a two-sample validation design was employed. Model testing was first conducted in Sample 1 (*N* = 2,096) and subsequently cross-validated in an independent Sample 2 (*N* = 1,004), allowing the stability of the measurement structure to be evaluated across large independent samples. This approach provides a rigorous test of whether the theoretically specified model generalizes beyond the initial sample without relying on sample-specific modifications. In contrast to exploratory approaches, which may yield sample-dependent factor structures, cross-validation supports the evaluation of structural stability while preserving the original measurement configuration required for cross-cultural validation.

Step 2 identified localized misfit and proposed a modified CFA model based on Sample 1. Modification indices were inspected, and residual covariances were introduced only when (a) MI values exceeded 50 and were considered in descending order, (b) the modification was theoretically interpretable as localized item dependence rather than substantive cross-loading, and (c) the confirmatory factor structure was preserved. These respecifications were intended to address localized measurement-level misfit while preserving the theoretically specified factor structure. The goal was not to alter the factor structure, but to account for localized item dependencies while maintaining the original measurement model. A maximum of three residual covariances were permitted, and cross-loadings were not introduced. After each respecification, factor loadings and inter-factor correlations were reviewed to ensure that the modifications did not alter the substantive interpretation of the latent factors. The modified model was subsequently evaluated through cross-validation in Sample 2 (Step 4), and only modifications demonstrating stable fit across samples were retained. Detailed justification for the specific item pairs is provided in section “3 Results.”

Step 3 compared competing models—including correlated-factor, higher-order, and bifactor models—using Sample 1 to determine the most appropriate structural representation. Step 4 cross-validated all models in Sample 2 to examine generalizability. Following the selection of the final model through model comparisons in Steps 3 and 4, Step 5 estimated internal consistency (Cronbach’s α and McDonald’s ω) using Sample 1, and Step 6 examined external validity in Sample 2 via correlations with related measures.

Model fit was evaluated using CFI, TLI, RMSEA, and SRMR, interpreted according to commonly cited guidelines: CFI/TLI ≥ 0.90 (≥0.95 preferred), RMSEA ≤ 0.08 (≤0.06 good), and SRMR ≤ 0.08 (Hu and Bentler, 1999; Kline, 2016). CFA models employed MLR estimation; this approach was selected because it provides robust standard errors under non-normality and is appropriate for large samples with four-category items treated as approximately continuous (Finney and DiStefano, 2013; Li, 2016; Muthén and Muthén, 2017). Test-retest reliability was examined using ICC(3,1) in Sample 3 for the total score and subscales, interpreted using Koo and Li (2016): <0.50 = poor, 0.50–0.75 = moderate, 0.75–0.90 = good, >0.90 = excellent. Data preparation was performed in SPSS 29. Confirmatory factor analyses were conducted in Mplus 8.8. Reliability and correlational analyses were carried out in R 4.3.2 using the *psych* package (version 2.5.1; psych::alpha, psych::omega) and the base cor function.

## 3 Results

### 3.1 Confirmatory factor analysis

The baseline model representing the original seven-factor correlated structure was first tested in Sample 1. The model demonstrated acceptable global fit (CFI = 0.923, TLI = 0.911, RMSEA = 0.048, SRMR = 0.041), and all factor loadings were statistically significant, ranging from 0.57 to 0.84. However, inspection of the factor correlation matrix revealed a very high correlation between Goal Setting and Planning and Implementing (*r* = 0.973), suggesting potential concerns regarding discriminant validity between these theoretically adjacent factors. Modification indices were primarily associated with two item pairs across these factors (items 5–6 and 19–20) and one pair within Adapting (items 21–28), indicating localized item dependence rather than misspecification of the overall dimensional structure.

The three residual covariances identified through the modification indices were theoretically interpretable. Items 5 (Goal Setting and Planning) and 6 (Implementing) both refer to closely related aspects of planning concrete steps toward career goals, resulting in partial content overlap beyond the variance captured by the latent Goal Setting and Planning factor. Similarly, Items 19 (Goal Setting and Planning) and 20 (Implementing) emphasize immediate action-taking behaviors in pursuing career objectives, which may produce shared variance attributable to similar wording and behavioral framing within the Implementing dimension. Finally, Items 21 and 28 both describe adaptive responses to unexpected career challenges, reflecting highly similar situational framing within the Adapting domain. None of these items involve reverse scoring. These localized dependencies therefore likely reflect minor wording or content overlap rather than misspecification of the underlying factor structure, and their replication in the independent validation sample (reported below) suggests that they reflect consistent linguistic or interpretive patterns rather than sample-specific overfitting.

A modified model was therefore estimated by freeing three theoretically interpretable residual covariances. After this respecification, model fit improved (CFI = 0.944, TLI = 0.935, RMSEA = 0.041, SRMR = 0.036; Table 3). Standardized factor loadings remained stable (0.55–0.84; Table 4), and the overall pattern of inter-factor relations was preserved, indicating that the modifications reduced measurement noise without altering the substantive meaning of the latent constructs. Notably, the correlation between Goal Setting and Planning and Implementing decreased to *r* = 0.888, which improved discriminant validity between the two theoretically related constructs (Table 5).

**TABLE 3:** Comparison of model fit indices for baseline, modified, higher-order, and bifactor models across samples.

Model	Parameters	AIC	BIC	aBIC	*χ^2^*	df	RMSEA	RMSEA 90% CI	CFI	TLI	SRMR
Sample 1 (N = 2,096)											
Baseline	105	102841.79	103434.81	103101.21	1904.01	329	0.048	[0.046, 0.050]	0.923	0.911	0.041
Modified	108	102294.83	102904.79	102561.66	1472.57	326	0.041	[0.039, 0.043]	0.944	0.935	0.036
Higher-order	94	102876.53	103407.43	103108.78	1938.94	340	0.047	[0.045, 0.049]	0.922	0.913	0.046
Bifactor	115	102616.31	103265.80	102900.43	1715.32	319	0.046	[0.044, 0.048]	0.931	0.919	0.042
Sample 2 (N = 1,004)											
Baseline	105	49952.47	50468.21	50134.72	1158.17	329	0.050	[0.047, 0.053]	0.912	0.898	0.047
Modified	108	49705.93	50236.40	49893.38	949.23	326	0.044	[0.040, 0.047]	0.934	0.923	0.043
Higher-order	94	49999.75	50461.46	50162.91	1213.02	340	0.051	[0.048, 0.054]	0.907	0.896	0.058
Bifactor	115	49871.91	50436.76	50071.51	1075.66	319	0.049	[0.045, 0.052]	0.919	0.904	0.053

AIC, Akaike information criterion; BIC, Bayesian information criterion; aBIC, sample-size adjusted BIC; *χ^2^*, Chi-square goodness-of-fit test; df, degrees of freedom; RMSEA, root mean square error of approximation; CI, confidence interval; CFI, comparative fit index; TLI, Tucker-Lewis index; SRMR, standardized root mean square residual. CFI, TLI, and RMSEA values reported are robust indices computed using the MLR estimator in Mplus.

**TABLE 4:** Item descriptives and standardized factor loadings for the modified model (Sample 1).

Item number	Factor	*M*	SD	Std. loading	SE	*z*
1	Hopefulness	2.79	0.77	0.74	0.01	56.24
8	Hopefulness	3.25	0.71	0.76	0.01	58.53
15	Hopefulness	3.15	0.77	0.84	0.01	82.13
22	Hopefulness	3.13	0.72	0.67	0.02	40.09
2	Self-reflection	3.31	0.63	0.61	0.02	29.11
9	Self-reflection	3.30	0.57	0.65	0.02	34.93
16	Self-reflection	3.27	0.63	0.70	0.02	37.81
23	Self-reflection	3.27	0.65	0.74	0.02	45.98
3	Self-clarity	2.97	0.77	0.75	0.01	52.11
10	Self-clarity	2.86	0.89	0.67	0.02	43.15
17	Self-clarity	2.96	0.78	0.81	0.01	65.73
24	Self-clarity	3.15	0.71	0.66	0.02	37.07
4	Visioning	3.43	0.66	0.69	0.02	41.02
11	Visioning	3.18	0.83	0.70	0.02	44.35
18	Visioning	3.31	0.63	0.67	0.02	40.48
25	Visioning	3.15	0.66	0.72	0.02	47.47
5	Goal setting and planning	2.88	0.84	0.60	0.02	33.00
12	Goal setting and planning	3.05	0.78	0.73	0.01	51.22
19	Goal setting and planning	3.05	0.80	0.67	0.02	38.94
26	Goal setting and planning	3.17	0.72	0.77	0.01	62.56
6	Implementing	3.06	0.73	0.68	0.02	39.80
13	Implementing	2.77	0.82	0.64	0.02	37.40
20	Implementing	3.12	0.68	0.76	0.01	54.93
27	Implementing	3.26	0.66	0.59	0.02	28.41
7	Adapting	3.09	0.78	0.69	0.02	45.54
14	Adapting	3.26	0.68	0.78	0.01	55.47
21	Adapting	3.32	0.62	0.55	0.02	26.89
28	Adapting	3.24	0.66	0.62	0.02	31.42

*M*, mean; SD, standard deviation; Std. loading, standardized factor loading from the modified model. All loadings are significant at *p* < 0.001. Sample 1 (*N* = 2,096).

**TABLE 5:** Inter-factor correlations: baseline (upper triangle) and modified (lower triangle) models, Sample 1.

Factor	1	2	3	4	5	6	7
1. Hopefulness	–	0.567	0.716	0.670	0.630	0.691	0.758
2. Self-reflection	0.338	–	0.586	0.782	0.644	0.634	0.713
3. Self-clarity	0.624	0.482	–	0.638	0.601	0.645	0.631
4. Visioning	0.517	0.590	0.552	–	0.755	0.653	0.665
5. Goal setting and planning	0.488	0.569	0.504	0.615	–	0.973	0.650
6. Implementing	0.618	0.544	0.555	0.556	0.888	–	0.733
7. Adapting	0.687	0.481	0.496	0.444	0.506	0.612	–

Upper triangle, baseline model; lower triangle, modified model (Sample 1, standardized estimates). The correlation between goal setting and planning and implementing decreased from 0.973 to 0.888 after freeing three residual covariances (items 5–6, 19–20, 21–28), indicating that part of the original association was attributable to localized item dependence.

The modified measurement model identified in Sample 1 was subsequently evaluated in the independent validation sample (Sample 2). As reported in Table 3, the model demonstrated comparable fit (CFI = 0.934, TLI = 0.923, RMSEA = 0.044, SRMR = 0.043), supporting the stability of the measurement structure and the localized residual dependencies across samples. To provide additional transparency regarding the empirical structure of the HAI, an exploratory factor analysis (EFA) was conducted on Sample 1. The results, including those from parallel analysis, are reported in the Supplementary Materials. However, because the HAI is a theory-driven instrument with a priori specified factor structure, the EFA results were not used to modify item–factor assignments or to redefine the underlying construct.

### 3.2 Model comparison: modified vs. higher-order vs. bifactor model

Because the modified model demonstrated superior fit relative to the baseline model, subsequent structural comparisons were conducted using the modified specification as the reference. The higher-order and bifactor models were therefore estimated with the same three residual covariances retained, ensuring that model comparisons reflected differences in higher-level structure rather than localized item misfit (Table 3).

**FIGURE 1:**
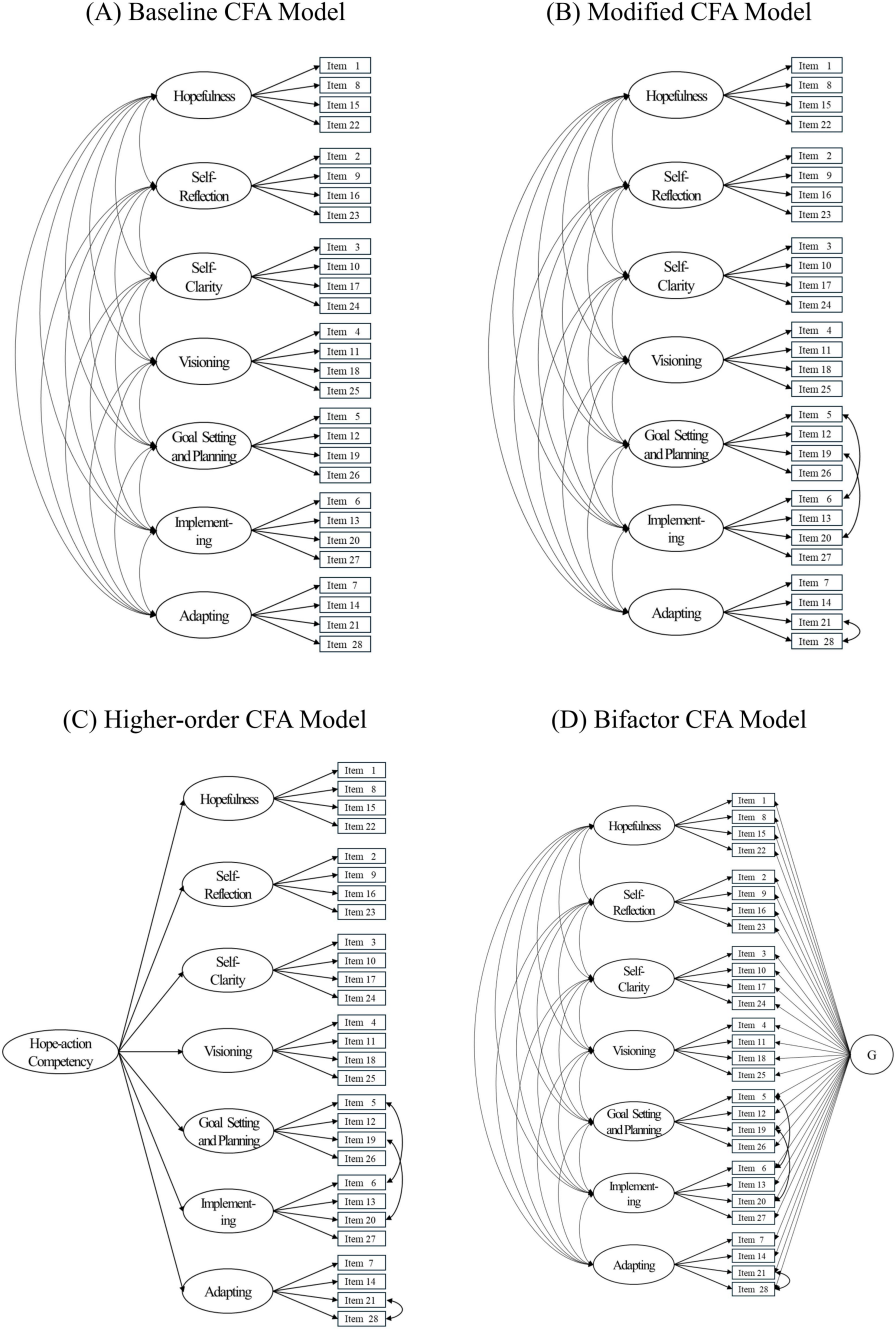
Comparison of confirmatory factor analysis (CFA) models of the Korean Hope-Action Inventory. **(A)** Baseline CFA model. **(B)** Modified CFA model. **(C)** Higher-order CFA model. **(D)** Bifactor CFA model.

Competing structural models—the baseline, modified, higher-order, and bifactor models—are depicted in Figure 1 and were compared using Sample 1. The modified model showed the most favorable and interpretable fit, outperforming the higher-order model (CFI = 0.922, TLI = 0.913, RMSEA = 0.047, SRMR = 0.046) and the bifactor model (CFI = 0.931, TLI = 0.919, RMSEA = 0.046, SRMR = 0.042). All competing models—the modified, higher-order, and bifactor models—were then subjected to cross-validation in Sample 2 to examine structural stability. The modified model again provided the best fit (CFI = 0.934, TLI = 0.923, RMSEA = 0.044, SRMR = 0.043), whereas the baseline (CFI = 0.912, TLI = 0.898, RMSEA = 0.050, SRMR = 0.047), higher-order (CFI = 0.907, TLI = 0.896, RMSEA = 0.051, SRMR = 0.058), and bifactor (CFI = 0.919, TLI = 0.904, RMSEA = 0.049, SRMR = 0.053) models showed weaker performance. These results confirmed the generalizability of the modified model across independent samples.

### 3.3 Reliability

Internal consistency of the Korean HAI was examined in Sample 1 using both Cronbach’s α and McDonald’s ω based on the modified model (Table 6). All subscales demonstrated acceptable to good reliability. Cronbach’s α coefficients ranged from 0.764 (Implementing) to 0.835 (Hope), and ω coefficients ranged from 0.742 (Adapting) to 0.842 (Hope). The total scale showed excellent internal consistency (α = 0.937, ω = 0.938). These results indicate that the Korean HAI subscales and overall score possess adequate reliability for research and applied use. Detailed reliability coefficients for each subscale are presented in Table 6.

**TABLE 6:** Cronbach’s α and McDonald’s ω for the modified model of Korean Hope-Action Inventory (HAI) (Sample 1).

Factor	Cronbach’s α	McDonald’s ω
Hopefulness	0.835	0.842
Self-reflection	0.767	0.772
Self-clarity	0.809	0.814
Visioning	0.785	0.788
Goal setting and planning	0.789	0.783
Implementing	0.764	0.759
Adapting	0.769	0.742
Total	0.937	0.938

Coefficients were estimated from Sample 1 (*N* = 2,096) based on the modified model.

Test-retest reliability was evaluated in Sample 3 (*N* = 50) across a 4-week interval. The total HAI score showed a high temporal stability (*r* = 0.81, *p* < 0.001; ICC = 0.79, 95% CI [0.66, 0.88]). At the subscale level, reliability coefficients ranged from *r* = 0.55 to 0.81 and ICC = 0.54 to 0.81 (see Table 7). According to the criteria proposed by Koo and Li (2016), the total score showed good test-retest reliability, whereas most subscales demonstrated moderate to good temporal stability.

**TABLE 7:** Test-retest reliability of the Korean Hope-Action Inventory (4-week interval, *N* = 50).

Scale	*r*	ICC	95% CI
Hopefulness	0.81	0.81	[0.682, 0.885]
Self-reflection	0.55	0.55	[0.324, 0.717]
Self-clarity	0.77	0.77	[0.629, 0.863]
Visioning	0.68	0.68	[0.501, 0.806]
Goal setting and planning	0.70	0.69	[0.518, 0.813]
Implementing	0.67	0.66	[0.466, 0.790]
Adapting	0.55	0.54	[0.308, 0.710]
HAI total	0.81	0.79	[0.661, 0.876]

*r*, Pearson correlation across a 4-week interval. ICC, intraclass correlation coefficient (single-measure, two-way mixed effects).

### 3.4 Convergent validity

Convergent validity of the Korean HAI was examined by analyzing its correlations with theoretically related constructs in Sample 2 (see Table 8). Overall, the pattern of correlations was consistent with the hypothesized relationships. The Hopefulness subscale showed the strongest associations with dispositional hope and optimism. Self-reflection was most strongly related to the self-reflectiveness component of the Assessment of Human Agency (AHA), consistent with its conceptual emphasis on reflective processing. Self-clarity showed its highest association with vocational identity, whereas Visioning was most strongly related to AHA forethought, reflecting its future-oriented nature. Goal Setting and Planning and Implementing demonstrated their strongest association with the agentic components of the AHA and with career goal and preparation competency. Adapting showed comparatively weaker associations with most constructs but was most strongly related to PHCI-Flex, consistent with its conceptual focus on adaptive flexibility. Finally, the HAI total score showed positive associations with vocational identity, human agency, dispositional hope, optimism, and career goal and preparation competency, and a negative association with emotional and personality-related career decision-making difficulties. These results provide overall support for the convergent validity of the Korean HAI.

**TABLE 8:** Correlations of Hope-Action Inventory (HAI) total and subscale scores with external validity indicators (Sample 2).

HAI	VI	AHA-IT	AHA-FT	AHA-RA	AHA-RF	K-DHS	LOT-R	CCS-Goal	VISA-Flex	PHCI-Flex	EPCD
Hopefulness	0.41	0.41	0.40	0.46	0.41	0.72	0.70	0.46	0.01^ns^	0.19	−0.57
Self-reflection	0.15	0.40	0.40	0.41	0.46	0.39	0.17	0.32	0.17	0.16	−0.09*
Self-clarity	0.45	0.38	0.41	0.46	0.46	0.57	0.40	0.41	-0.03 ^ns^	0.12	−0.48
Visioning	0.28	0.41	0.73	0.47	0.47	0.42	0.29	0.41	0.01 ^ns^	0.09*	−0.21
Goal setting and planning	0.30	0.64	0.48	0.68	0.46	0.51	0.23	0.66	0.01 ^ns^	0.11	−0.26
Implementing	0.34	0.65	0.37	0.67	0.45	0.60	0.32	0.67	0.01 ^ns^	0.09*	−0.34
Adapting	0.25	0.35	0.32	0.41	0.38	0.58	0.39	0.34	0.19	0.44	−0.32
Overall	0.45	0.65	0.62	0.71	0.61	0.75	0.51	0.65	0.07*	0.24	−0.46

Correlations are Pearson coefficients. Overall represents the HAI total score. VI, Vocational Identity; AHA, Assessment of Human Agency (IT, intentionality, FT, forethought, RA, self-reactiveness, RF, self-reflectiveness); K-DHS, Korean Dispositional Hope Scale; LOT-R, Life Orientation Test-Revised; CCS-Goal, Career Competencies Scale—Goal Setting and Preparation; VISA-Flex, Vocational Identity Status Assessment—Flexibility; PHCI-Flex, Planned Happenstance Career Inventory—Flexibility; EPCD, Emotional and Personality-related Career Decision-making Difficulties. All correlations were significant at *p* < 0.001 unless otherwise indicated (*p < 0.01; ns, not significant). *N* = 1,004.

## 4 Discussion

The present study aimed to evaluate the psychometric properties of the HAI among Korean university students by examining its factorial structure, reliability, and convergent validity. Using two large independent samples, we compared correlated, hierarchical, and bifactor representations and found that a modified seven-factor correlated model (the modified model) provided the most appropriate and replicable representation of hope-action competencies in the Korean cultural context. Overall, the findings support the use of the Korean HAI as a reliable and theoretically meaningful assessment tool while also offering several conceptual and practical insights.

### 4.1 Factorial structure and conceptual interpretation

A primary objective of this study was to determine the most appropriate psychometric representation of hope-action competencies. In line with the findings of Santilli et al. (2021), which favored a correlated representation, but differing from studies that supported hierarchical structures (e.g., Schreiber et al., 2013; Currie et al., 2024), the present findings indicated that a correlated-factor approach provided the most adequate description of the Korean data. The original baseline model, similar to that employed in previous validations, demonstrated acceptable fit while also revealing a notably high association between Goal Setting and Planning and Implementing, underscoring the need to carefully evaluate alternative representations. Based on the predefined fit criteria (CFI/TLI ≥ 0.90, RMSEA ≤ 0.08, SRMR ≤ 0.08; Hu and Bentler, 1999; Kline, 2016), all competing models satisfied the minimum standards for acceptable fit; nevertheless, the modified correlated model was the only model that consistently demonstrated “good” fit across both samples (RMSEA ≤ 0.06, SRMR ≤ 0.08, CFI/TLI approaching 0.95), whereas the hierarchical and bifactor models yielded comparatively weaker or marginal indices, particularly in Sample 2. Importantly, this conclusion was derived from analyses of two large and independent samples, providing substantial statistical power and enabling rigorous cross-validation.

This pattern implies that, for Korean university students, hope-action competencies are better conceptualized as a set of interrelated but distinguishable action competencies rather than as indicators of a single overarching factor. The result aligns with the Italian findings (Santilli et al., 2021) and contrasts with the hierarchical solution reported in a Canadian clinical sample (Currie et al., 2024). Such discrepancies suggest that the psychometric representation of the HAI may be context-sensitive, influenced by developmental stage and cultural understandings of agency.

The need for limited model respecification was examined through both statistical evidence and substantive considerations. Examination of modification indices in Sample 1 indicated several areas of localized residual dependencies at the item level. Three residual covariances were introduced only when they were theoretically interpretable as localized item dependence associated with translation and linguistic wording effects, rather than as evidence of misspecification of the underlying factor structure. Two covariances were specified between Goal Setting and Planning and Implementing items because the Korean wording appeared to activate overlapping self-regulatory scripts that may blur the planning–execution distinction at the item level. Specifically, “setting deadlines to complete goals” (Item 5, Goal Setting and Planning) and “keeping myself focused to complete plans” (Item 6, Implementing) were likely perceived by Korean students as behaviorally adjacent actions involved in managing and completing planned goals, which may generate shared variance at the item level beyond that explained by the latent factors. Similarly, “making a list of things to complete” (Item 19, Goal Setting and Planning) and “taking the next steps to meet goals” (Item 20, Implementing) may be interpreted as closely related steps involved in organizing and progressing through goal-related tasks, which may also produce shared variance at the item level beyond the variance explained by the latent factors. Such residual associations are consistent with established CFA practice, which notes that correlated errors may occur not only among indicators within the same factor but also across factors when items are very similarly worded or share similar linguistic framing, and therefore contain variance beyond that explained by the latent constructs (Brown, 2015). These patterns suggest that the localized residual covariances are more appropriately interpreted as translation- and wording-related measurement effects rather than as evidence of misspecification of the theoretical factor structure.

One additional covariance was specified within Adapting between “being open to changing plans when necessary” (Item 21) and “being prepared to change if the situation changes” (Item 28), which are near-paraphrastic expressions of flexibility in the Korean translation and may therefore generate additional shared variance at the item level beyond the common Adapting factor. These respecifications were limited to three pairs and were replicated in Sample 2, supporting the stability of the modified correlated model. The fact that identical dependencies emerged in the independent sample suggests that these residual associations reflect stable linguistic and translation-related measurement effects, rather than sample-specific overfitting or *post hoc* model correction. This finding further suggests that the localized dependencies arise from how flexibility-related behaviors are expressed in Korean, rather than from problems with the underlying Adapting construct itself.

Taken together, the results of the present study provide evidence that the Korean version of the HAI retains the multidimensional logic proposed by Hope-Action Theory, while also indicating the need for minor measurement-level adjustments to the original model. Across two large and independent samples, a modified seven-factor correlated model emerged as the most stable and well-fitting representation. Consistent with this model selection, interpretation of the Korean HAI is most appropriately conducted at the level of its subscales, reflecting the multidimensional and correlated nature of hope-action competencies. Overall, these findings suggest that the observed model refinements are more appropriately understood as measurement-level adjustments related to translation and linguistic representation, rather than as changes to the underlying theoretical structure of the HAI.

### 4.2 Reliability and convergent validity

Consistent with previous studies conducted in North American and European contexts (Amundson et al., 2018; Clarke et al., 2018; Santilli et al., 2021; Schreiber et al., 2013), the Korean HAI demonstrated strong internal consistency at the total-score level, supporting the robustness of hope-action competencies as a multidimensional configuration of interrelated competencies across cultural settings. Subscale reliabilities were also within acceptable to good ranges, indicating that the Korean HAI items function coherently within each domain among Korean university students.

Importantly, the pattern of subscale reliability observed in the present study should be interpreted in light of prior validation research using earlier versions of the HAI. In studies employing the original item set, the Self-reflection subscale consistently showed lower and more variable internal consistency relative to other dimensions across North American and European samples (Santilli et al., 2021; Schreiber et al., 2013; Yoon et al., 2015). In contrast, other subscales generally demonstrated acceptable but comparatively modest reliability. These findings suggest that subscale-level variability was a recurring feature of the earlier instrument versions.

The present study found that Self-reflection demonstrated acceptable internal consistency comparable to that of other HAI subscales. This pattern closely parallels findings from the recent Canadian validation of the HAI, which reported consistently high reliabilities across all subscales (Currie et al., 2024). Taken together, these findings suggest that the lower reliability previously observed for Self-reflection is more plausibly attributable to characteristics of the earlier HCCI item set, and that the HAI items used in the present study and by Currie et al. (2024) yield more stable measurement of this dimension across the cultural contexts examined to date. Overall, these results support the reliability of the Korean HAI and indicate that its subscales provide sufficiently consistent measurement for both research and applied use.

Convergent validity analyses further extend prior research by providing subscale-level evidence of construct validity. Whereas earlier studies primarily examined convergent validity at the level of the HAI total score (e.g., Niles et al., 2010; Santilli et al., 2021), the present findings demonstrate differentiated patterns of association between specific HAI dimensions and theoretically corresponding external constructs. As hypothesized, Self-reflection was most strongly related to reflective agency, Self-clarity aligned most closely with vocational identity, and Visioning corresponded with future-oriented agency. Goal Setting and Planning and Implementing were most strongly associated with agentic action processes and career goal and preparation competency, reflecting their shared emphasis on goal-directed behavior. In addition, Adapting demonstrated its strongest associations with flexibility-related constructs, consistent with its conceptual focus on adaptive flexibility.

At the total-score level, the Korean HAI demonstrated strong positive associations with dispositional hope, optimism, human agency components, and career goal and preparation competency, as well as a negative association with emotional and personality-related career decision-making difficulties. This pattern closely mirrors prior findings from North American and European studies, which reported robust associations between the HAI total score and dispositional hope, agency-related constructs, and adaptive career functioning (e.g., Currie et al., 2024; Niles et al., 2010; Santilli et al., 2021). Taken together, these findings indicate that the Korean HAI not only replicates key reliability and convergent validity patterns reported in previous studies but also extends the evidence base by demonstrating differentiated subscale-level validity within a Korean university student population.

### 4.3 Theoretical and practical implications

The present findings provide cross-cultural support for conceptualizing hope-action competency as a multidimensional construct composed of correlated but distinguishable components. Although hierarchical and bifactor models also demonstrated acceptable fit, the consistently superior performance of the modified correlated-factor model across two large independent samples suggests that core hope-action processes are best understood as interacting competencies rather than as indicators of a single higher-order factor. This result aligns with prior Italian findings (Santilli et al., 2021) and highlights the context-sensitive nature of the HAI’s factor structure.

In addition, the present study extends previous validation research by demonstrating differentiated convergent validity at the subscale level. Whereas earlier studies focused primarily on total-score validity, the current findings show that specific HAI dimensions converge meaningfully with theoretically corresponding constructs related to vocational identity, human agency, career goal and preparation competency, and flexibility.

From a practical perspective, the Korean HAI demonstrates adequate reliability and validity to support its use in both research and applied settings. Interpretation at the subscale level may be particularly informative for identifying specific strengths and needs in career development processes. At the same time, despite the superiority of the correlated-factor model, the Korean HAI total score may serve as a pragmatic global indicator of hope-action competency when a broad, summary-level assessment is required, such as in large-scale research or program evaluation.

Taken together, the present study contributes to the literature by providing one of the first comprehensive psychometric evaluations of the Hope-Action Inventory (HAI) in a non-North American context. By examining the scale’s factorial structure and validity in two large independent Korean samples, the findings extend the evidence base for Hope-Action Theory and support the cross-cultural applicability of the HAI. These results suggest that the Korean HAI can serve as a reliable and theory-grounded instrument for assessing hope-action competencies among university students.

### 4.4 Limitations and future directions

Several limitations should be considered when interpreting the present findings. First, although the factorial structure and convergent validity of the Korean HAI were evaluated using two large and independent samples, the participants were limited to Korean university students. Consequently, the generalizability of the results to other age groups, educational levels, and occupational or clinical populations remains to be established. Future studies should examine the psychometric properties of the Korean HAI across more diverse samples and contexts.

Second, while the modified seven-factor correlated model demonstrated the most stable and optimal fit, the hierarchical and bifactor models also yielded acceptable overall fit indices. This indicates that alternative structural representations of hope-action competency cannot be definitively ruled out, and that the most appropriate level of abstraction may vary depending on research purpose and sample characteristics. Longitudinal, multi-group, and intervention-based studies may help clarify whether and under what conditions a higher-order hope-action factor becomes salient.

Third, model refinement involved the specification of three residual covariances, corresponding to six items primarily related to goal setting and planning, implementation, and adapting processes. Although these residual associations were theoretically interpretable, limited in number, and replicated across independent samples, it should be noted that the use of modification indices has an exploratory component, as modification indices are data-driven and may reflect sample-specific response patterns if applied without strong theoretical justification (Brown, 2015). In the present study, however, residual covariances were introduced only when they were theoretically interpretable as translation- and wording-related localized item dependence and when the same residual relationships were replicated in an independent sample. These findings suggested that minor wording refinements may help reduce linguistic overlap between items. Future research should consider refining these items and re-examining the overall factor structure of the Korean HAI following revision of these items.

Despite these limitations, the present study provides strong evidence for the reliability and convergent validity of the Korean HAI. The current version of the instrument demonstrates sufficient psychometric adequacy to support its use in both research and applied settings, particularly when interpretation is guided by its multidimensional and correlated subscale structure.

## Data Availability

The raw data supporting the conclusions of this article will be made available from the corresponding author on reasonable request.

## Supplementary material

Supplementary Appendix A Korean HAI Items.
