# Supplementary material for: Psychometric validation of the Korean Hope-Action Inventory among university students
Source: Front Psychol. 2026 May 18;17:1794159. doi: 10.3389/fpsyg.2026.1794159 (PMC13223180; doi:10.3389/fpsyg.2026.1794159)
Supplement: Supplementary file 1 [file Table_1.docx]

**Supplementary Analysis: Exploratory Factor Analysis of the Hope–Action Inventory (Sample 1)**

To enhance transparency regarding the empirical factor structure of the Hope–Action Inventory (HAI), an exploratory factor analysis (EFA) was conducted on Sample 1. The analysis was based on Sample 1 (*N* = 2,096) and included 28 items of the HAI. Prior to the EFA, the adequacy of the data for factor analysis was examined. The Kaiser–Meyer–Olkin (KMO) measure of sampling adequacy was .95, indicating excellent suitability for factor analysis. Bartlett’s test of sphericity was significant, *χ*²(378) = 26,919.25, *p* < .001, confirming that the correlation matrix was appropriate for factor analysis. EFA was performed using principal axis factoring with oblimin rotation. To determine the number of factors, a parallel analysis (100 iterations) was conducted by comparing the observed eigenvalues with those obtained from randomly generated data. The results of the parallel analysis suggested a seven-factor solution.

The seven-factor solution closely reflected the theoretical structure of the HAI, with items loading primarily on their intended factors. This pattern indicates that the hypothesized multidimensional structure of the HAI is well supported at the empirical level.

For transparency, the factor loadings of the seven-factor solution are reported in Table S1. Although the overall structure was clearly reproduced, a small degree of overlap was observed between the Goal Setting and Planning and Implementing factors, as indicated by minor cross-loadings and moderate correlations between these factors. Importantly, this empirical overlap is consistent with the residual covariances that were specified between planning- and implementation-related items in the confirmatory factor analysis of Sample 1 (e.g., items 5–6 and 19–20). We interpret this convergence between the EFA and CFA findings as evidence of localized item dependence within goal-directed action processes rather than as misspecification of the theoretical factor structure, supporting the retention of the seven-factor theoretical model.

At the item level, a small number of deviations from the expected loading pattern were observed. For example, Item 20 showed a relatively stronger association with the Goal Setting and Planning factor than with its theoretically assigned Implementing factor, and Item 27 showed a relatively modest loading on the Implementing factor. These patterns are consistent with the localized overlap between planning and implementation processes described above, rather than indicating broader structural inconsistency. Overall, the EFA results provided strong support for the theoretical seven-factor structure of the HAI.

Table S1. Exploratory Factor Analysis of the 28 HAI Items (Sample 1, *N* = 2,096)

| Item # | Hopefulness | Self-Reflection | Self-Clarity | Visioning | Goal Setting and Planning | Implementing | Adapting |
| --- | --- | --- | --- | --- | --- | --- | --- |
| 1 | .68 |  |  |  |  |  |  |
| 8 | .63 |  |  |  |  |  |  |
| 15 | .82 |  |  |  |  |  |  |
| 22 | .37 |  |  |  |  |  |  |
| 2 |  | .56 |  |  |  |  |  |
| 9 |  | .44 |  |  |  |  |  |
| 16 |  | .54 |  |  |  |  |  |
| 23 |  | .72 |  |  |  |  |  |
| 3 |  |  | .69 |  |  |  |  |
| 10 |  |  | .52 |  |  |  |  |
| 17 |  |  | .83 |  |  |  |  |
| 24 |  |  | .65 |  |  |  |  |
| 4 |  |  |  | .57 |  |  |  |
| 11 |  |  |  | .72 |  |  |  |
| 18 |  |  |  | .41 |  |  |  |
| 25 |  |  |  | .45 |  |  |  |
| 5 |  |  |  |  | .51 |  |  |
| 12 |  |  |  |  | .53 |  |  |
| 19 |  |  |  |  | .78 |  |  |
| 26 |  |  |  |  | .54 |  |  |
| 6 |  |  |  |  |  | .87 |  |
| 13 |  |  |  |  |  | .39 |  |
| 20 |  |  |  |  |  | .67 |  |
| 27 |  |  |  |  |  | .31 |  |
| 7 |  |  |  |  |  |  | .32 |
| 14 |  |  |  |  |  |  | .49 |
| 21 |  |  |  |  |  |  | .66 |
| 28 |  |  |  |  |  |  | .68 |

*Note.* Factor loadings ≥ .30 are presented. Extraction method = principal axis factoring; rotation = oblimin. Parallel analysis supported a seven-factor solution. Item numbers correspond to the English version of the HAI.
