# Supplementary material for: Psychometric validation of the Korean Hope-Action Inventory among university students
Source: Front Psychol. 2026 May 18;17:1794159. doi: 10.3389/fpsyg.2026.1794159 (PMC13223180; doi:10.3389/fpsyg.2026.1794159)
Supplement: Supplementary Appendix A — Korean HAI Items. [file Supplementary_file_1.docx]

**Appendix A**

Sample English and Korean Items from the Hope-Action Inventory (HAI)

The HAI is a proprietary instrument. The following sample items are provided solely to illustrate translation fidelity and face validity. Items are rated on a 4-point Likert scale (1 = Not at all like me; 4 = Very much like me).

| **Subscales** | **English Sample Items** | **Korean Sample Items** |
| --- | --- | --- |
| **Hopefulness** | I am hopeful when I think about my future. | 나는 내 미래에 대해 생각하면 희망차다. |
|  | I believe my dreams will come true. | 나는 내 꿈이 이루어질 것이라고 믿는다. |
|  | I think positively about my future. | 나는 내 미래에 대해 긍정적으로 생각한다. |
| **Self-Reflection** | I think about things that have happened to me. | 나는 나에게 일어났었던 일들에 대해 생각해 본다. |
|  | I think about what is the common theme among the things I like. | 나는 내가 좋아하는 것들 간의 공통점이 무엇인지 생각해 본다. |
|  | I think about how my personal experiences influence my decisions in life. | 나는 나의 개인적인 경험들이 삶에서의 내 의사결정에 어떻게 영향을 미치는지 생각한다. |
| **Self-Clarity** | I can describe who I am. | 나는 내가 누구인지를 설명할 수 있다. |
|  | I can list at least five things that I am good at. | 나는 내가 잘 하는 것을 적어도 다섯 개는 열거할 수 있다. |
|  | I am clear about who I am. | 나는 내가 누구인지에 대해 분명하다. |
| **Visioning** | I often dream about my future. | 나는 종종 내 미래에 대해 꿈꾸어 본다. |
|  | I often envision my future 2, 5, or 10 years from now. | 나는 종종 2년, 5년, 또는 10년 후 나의 미래를 상상하곤 한다. |
|  | I often imagine possible future events in my life. | 나는 종종 내 삶에서 일어날 가능성이 있는 미래의 사건들을 상상하곤 한다. |
| **Goal Setting and Planning** | I set deadlines to complete my goals. | 나는 나의 목표를 달성하기 위해 마감일을 설정한다. |
|  | I often list things that I need to do to reach my goals. | 나는 종종 내 목표에 도달하기 위해 해야 할 것들을 열거해 본다. |
|  | I make a list of things that I want to complete. | 나는 내가 완료하고자 하는 것들의 목록을 만든다. |
| **Implementing** | I keep myself focused so that I can complete my plans. | 나는 나의 계획을 마무리할 수 있도록 집중한다. |
|  | I work hard to meet my goals even when there are distractions. | 나는 나를 산만하게 하는 것들이 있을 때조차 내 목표를 성취하기 위해 열심히 일한다. |
|  | I take the next steps to meet my goals. | 나는 내 목표를 달성하기 위한 다음 단계들을 밟는다. |
| **Adapting** | I am willing to try new experiences that might help me to achieve my goals. | 내 목표 달성에 도움이 될 수도 있는 새로운 경험을 기꺼이 시도한다. |
|  | I am open to change that might improve my chance to reach my goals. | 내 목표에 도달할 가능성을 높여줄지 모르는 변화에 대해 열린 자세를 가지고 있다. |
|  | I am open to making changes to my plans when necessary. | 나는 필요 시 내 계획을 변경하는 것에 대해 열려있다. |

*Note*. Three sample items are shown per subscale for illustrative purposes only. The full HAI item set is proprietary and not publicly available. Korean translations followed established back-translation procedures (Beaton et al., 2000).
